# Gastric *Helicobacter pylori* infection associates with an increased risk of colorectal polyps in African Americans

**DOI:** 10.1186/1471-2407-14-296

**Published:** 2014-04-28

**Authors:** Hassan Brim, Marwah Zahaf, Adeyinka O Laiyemo, Mehdi Nouraie, Guillermo I Pérez-Pérez, Duane T Smoot, Edward Lee, Hadie Razjouyan, Hassan Ashktorab

**Affiliations:** 1 Department of Pathology, Howard University, Washington, DC, USA; 2 Department of Medicine and Cancer Center, Howard University, Washington, DC, USA; 3 Sickle Cell Disease Center, Department of Medicine, Howard University, Washington, DC, USA; 4 Department of Medicine and Microbiology, New York University, New York, NY, USA; 5 Cancer Center and Department of Medicine, Howard University College of Medicine, 2041 Georgia Avenue, NW, Washington, DC 20060, USA; 6 Department of Medicine, Monmouth Medical Center, Drexel University College of Medicine, Long Branch, NJ 07744, USA

**Keywords:** African Americans, *H. pylori* infection, Colorectal neoplasm, Gastric lesion, Risk factors, Forty year and older

## Abstract

**Background:**

Gastric *Helicobacter pylori* (*H. pylori*) infection and colorectal polyps are more prevalent in African Americans than in the general population. We aimed to investigate whether gastric *H. pylori* infection is associated with colorectal polyps in African Americans.

**Methods:**

Medical records of African Americans, 40 years and older (n = 1256) who underwent bidirectional gastrointestinal endoscopy on the same day were reviewed. *H. pylori* status was assessed by immunohistochemistry on gastric specimens. Colorectal polyps were confirmed by histological examination of colorectal biopsies. A subset of serum samples from healthy and polyp-bearing patients (n = 163) were analyzed by ELISA for anti-*H. pylori* and anti-CagA antibodies. The crude and adjusted effect of *H. pylori* on the risk of colorectal adenoma and polyp were computed by logistic regression models.

**Results:**

The prevalence of colorectal polyps and adenomas were 456 (36%) and 300 (24%) respectively. Colorectal polyps were more prevalent in gastric *H. pylori* infected than non-infected subjects [43% vs. 34%; Odds Ratio (OR) (95% CI): 1.5 (1.2-1.9), *P* = 0.001]. Patients with *H. pylori-*associated chronic active gastritis were at high risk to have adenomas [Unadjusted OR (95% CI): 1.3 (1.0-1.8); *P* = 0.04]. There was no difference in histopathology, size, or location of polyps with respect to *H. pylori* status. Gastric *H. pylori* infection, age, male gender and high risk clinical presentations were independent risk factors for colorectal polyps. Serological testing also revealed a higher prevalence of *H. pylori* and its toxin Cag-A in polyp patients vs. non polyp patients’ sera, although in a non-statistically significant manner.

**Conclusions:**

This study showed that current gastric *H. pylori* infection is associated with an increased risk of colorectal polyps in African Americans. Patients with *H. pylori* induced gastritis may benefit from early screening colonoscopy as a preventative measure for colorectal cancer.

## Background

Colorectal cancer (CRC) is the third most common cancer and the third most common cause of cancer deaths in both men and women in US [1]. In its sporadic form, CRC mostly arises from adenomatous polyps (adenomas). CRC can also arise from hyperplastic polyps [2,3]. Early detection and removal of colorectal polyps have led to a decrease in the incidence and mortality from CRC [4-6]. Recent interest have been directed toward CRC prevention and the possible role of infectious agents in the polyp to cancer sequence [7-10]. For instance, many epidemiological studies have linked *H. pylori’*s infection to colorectal neoplasm either through high prevalence of *H. pylori* seropositivity among CRC or colorectal polyp patients [11-14], or through the presence of bacterial byproducts and their trophic effects on colon mucosa [15-18], while others disagree [19-22]. Moreover, few studies have linked current *H. pylori* in the stomach [23] or colon [24-29] with colon cancer and/or polyps.

It is well known that *H. pylori* predisposes to the development of gastric cancer precursor lesions, thus it has been classified as class 1 carcinogen [30]. A recent publication by Sonneberg et al. revealed a wide range of effects of gastric *H. pylori* on the gastrointestinal tract with diseases that are inversely associated with *H. pylori*, such as reflux disease, erosive oesophagitis, Barrett’s oesophagus, and oesophageal adenocarcinoma, showing a striking rise during the recent decline of *H. pylori* infection in the general population [31]. Whether *H. pylori’s* effect on gastric mucosa predicts its effect on colon mucosa is still controversial. Indeed, a recent meta-analysis of the correlation between *H. pylori* and extra-gastric malignancies revealed a modest statistically significant relationship of *H. pylori* infection with both colon cancer and polyps [32]. *H. pylori’s* infection and colorectal lesions appear to be more common in African Americans compared to the Caucasian population in the US [1,33]. We sought to determine whether current gastric *H. pylori* infection was associated with the presence of colorectal polyps in a population at high risk for colorectal lesions.

## Methods

### Patients’ selection

We retrospectively reviewed the medical records of 1920 patients of which 1256 were included in the present study. The 1256 retained records correspond to African American patients, 40 years and older who underwent bidirectional endoscopy (complete colonoscopy and gastroscopy) at the same day from January 2005 to August 2009. The study was conducted at Howard University Hospital, a tertiary hospital serving predominantly African Americans in the District of Columbia, USA. The study was approved by the Howard University Hospital Institutional Review Board and we obtained consent from patients who provided blood samples for the serological analysis. Demographic variables included gender, race and age. Clinical and pathological data were collected with respect to reasons for undergoing bidirectional endoscopies, *H. pylori* immunohistochemistry (IHC) status of gastric biopsies, histo-pathological diagnosis of gastric specimens, and colorectal polyps’ type, size, grade of dysplasia and location. We divided our patients into high and average risk for colorectal polyps based on their presentations [34,35]. High risk patients were those with lower gastrointestinal (GI) blood loss, abdominal mass [34,35], and/or family/personal history of colorectal polyps or cancer [36]. Average risk patients were either asymptomatic and undergoing screening colonoscopy or suffered abdominal pain, epigastric pain unresponsive to treatment, acid peptic symptoms, change in bowel habits, weight loss or anemia. Patients were excluded if they had inflammatory bowel disease, malignancies including colorectal cancer, suboptimal bowel preparations, incomplete colonoscopies, and lack of data regarding *H. pylori* immunohistochemistry examination of gastric biopsies.

### Specimens

Gastric biopsies were taken during gastroscopy and were labeled as antrum, body, and fundus. Both gastric biopsies and colorectal polyps (when encountered) were harvested by biopsy, snare, piecemeal excision, or saline assisted endoscopic mucosal resection. Colorectal polyps were divided by location. Polyps located in cecum, ascending, and transverse colon were classified as “right sided”. Those located in descending colon, sigmoid, and rectum were classified as “left sided”. Patients with multiple polyps all over the colon were classified as having “both” right and left colon polyps. All specimens were sent to the pathology department after immersion in formalin. The colorectal polyps size was measured after tissue fixation. *H. pylori* status was identified using immunohistochemistry staining on gastric biopsies. A Novocastra Liquid mouse monoclonal Anti-*H. pylori* antibody was used (NCL-L-*H. pylori*, Clone#ULC3R, Leica Biosystems). An experienced gastrointestinal pathologist examined the specimens and made the histo-pathological classification of gastric biopsies and colorectal polyps. We classified *H. pylori* associated gastric lesions (independently of their distribution or severity) into chronic active (non-atrophic) gastritis, chronic atrophic gastritis with intestinal metaplasia, reactive gastropathy with foveolar hyperplasia, hyperplastic gastropathy and normal gastric mucosa [37]. We excluded gastric dysplasia and gastric cancers from our study. Colorectal polyps included hyperplastic (non-neoplastic) polyps and adenomatous (neoplastic) polyps. Adenomatous polyps were divided into advanced adenomas (tubular adenoma ≥1 cm, adenoma with > 25% villous component, and/or high grade dysplasia), and non-advanced adenoma (tubular adenoma <1 cm) [6].

### Serological tests for the detection of anti-*H. pylori* and anti-Cag-A in patients’ sera

We determined the presence of anti-*H. pylori* antibodies in serum using 96-well plates coated with *H. pylori* whole cell antigens, according to previously described methods [38,39]. All samples, standards and controls were run in duplicate. Serum samples (n = 163) were diluted 1:800 and incubated in the 96-well plate for 1 hr at 37°C. After washing the plates twice using EL × 50 Automated strip washer (Bio-Tek Instruments, Winooski VT), bound antibodies were detected by anti-human IgG linked to horseradish peroxidase (Biosource International, Camarillo, CA) diluted 1:4000 and incubated for 1 hr at 37°C. After washing, color development is produced by 2,2′-Azino-bis(3-Ethylbenzthiazoline-6-sulfonic acid) (Sigma Chemical Co. St Louis MO). Color in wells was analyzed in a MRX Revelation microplate reader (Dynex technologies INC, Chantilly, VA) at 450 nm within 30 min. All assays were performed with 4 positive and 2 negative controls. A positive result is one with an OD (optical density) ratio greater than one, as previously reported [40,41]. The specificity of this assay is 93.5% and its sensitivity is 99.4% [40,42].

For the detection of antibodies against CagA, 96-well plates coated with recombinant CagA protein prepared according to specifications previously reported [43,44]. All samples, were run in duplicate. Serum samples were diluted 1:100 and applied to wells and incubated for 1 hr at 37°C. Plates were washed twice and bound antibodies were detected by anti-human IgG linked to horseradish peroxidase (Biosource). The reporter for bound enzyme is horseradish peroxidase that was detected as mentioned above. Color is read in a microplate reader (Dynex) at 450 nm. Samples with OD ratio values greater than a pre-established cut-off (OD ratio 450 = 0.350) are considered positive as previously reported [44]. This assay has a specificity of 97% and a sensitivity of 96% [43]. Positive and negative controls were sera from biopsy- and culture-validated individuals obtained locally, in addition to positive and negative controls obtained from previous studies [38].

### Statistical analysis

We used Student’s *t*-test to compare the distribution of continuous variables between *H. pylori* positive and negative subjects. For categorical variables, we used Chi-square test. Then we computed unadjusted Odds Ratio (OR) (95% Confidence Interval [CI]) for potential predictors of colorectal polyps and adenomas. Multivariate logistic regression analysis was applied to compute the adjusted OR (95% CI) for predictors of colorectal polyps and adenomas. All variables with P < 0.2 from bivariate analysis were selected for multivariate logistic regression. The final model was developed with a stepwise backward approach. All variables with P value < 0.05 were considered statistically significant and remained in final model. All analyses were done using SPSS 17.0 (SPSS Inc., IL).

## Results

### Population and clinicopathological characteristics

Among 1920 potential participants, 1256 African Americans, aged forty or above, were eligible for this study. Non-African American patients (n = 100), as well as patients without bidirectional endoscopy (n = 385), patients with inflammatory bowel disease (n = 146) or lacking the *H. pylori* IHC stain (n = 33) were excluded. The prevalence of *H. pylori* infection was 366/1256 (29.1%) while the prevalence of colorectal polyps and adenomas were 456 (36%) and 300 (24%) respectively. The frequency of males was 433 (34%). The frequency of colorectal adenomas increased with age (from 15% in patients younger than 50 to 33% in those 70 years and older, p < 0.001). The same trend was observed for polyps (from 30% in patients younger than 50 to 39% in those 70 years and older, P = 0.033). The mean age (SD) 57 years (9.6) was the same for *H. pylori* infected and non-infected subjects (p = 0.9). The frequency of male gender in *H. pylori* negative and positive patients were 32% and 41%, respectively (p = 0.004). The frequency of chronic active gastritis (p <0.001) and colon polyps (p = 0.001) were higher in *H. pylori* positive patients. Polyp histology, size and location were not correlated to *H. pylori* status (Table 1). Colorectal polyps were located mainly in the left colon (54%). The same distribution was observed for adenomatous and hyperplastic polyps. Out of 1256 patients, 158 (13%) had personal or family history of colorectal polyps or cancer. Of these 158 subjects, 63 (40%) were males, 32 (20%) were *H. pylori* positive, and 77 (49%) had colorectal polyps.

**Table 1:** **Distribution of clinical variables by *H. pylori *** **status**

	***H. pylori *** **positive N = 366**	***H. pylori *** **negative N = 890**	***P *** **value**
Gastric lesions			
- No gastric lesion, n (%)	8 (2)	691 (78)	<0.001
- Chronic Active Gastritis, n (%)	305 (83)	62 (7)	
- Intestinal metaplasia, n (%)	46 (13)	98 (11)	
- Other lesions, n (%)	7 (2)	35 (4)	
Polyp, no (%)^1^	158 (43)	298 (34)	0.001
Polyp size in cm, mean (SD)^1^*	0.2 (0.15)	0.2 (0.15)	0.2
Polyp Histology*			
- Advanced Adenoma, n (%)	21 (13)	32 (11)	0.6
- Non-advanced adenoma, n (%)	87 (55)	160 (54)	
- Hyperplastic polyp, n (%)	50 (32)	106 (36)	
Polyp Location^1^*			
- Right side, n (%)	51 (32)	95 (32)	0.9
- Left side, n (%)	84 (53)	164 (55)	
- Both sides, n (%)	23 (15)	38 (13)	

^1^“Polyp” label in this table includes adenoma and hyperplastic polyp.

*Among subjects with polyps.

By univariate analysis, older age (p <0.001), male gender (p < 0.001), *H. pylori* positivity (p = 0.003) and chronic active gastritis (p = 0.04) were significantly associated with higher frequency of adenoma (Table 2.a). In a separate analysis, male gender and *H. pylori* were associated with higher frequency of polyps (Table 2.b).

**Table 2:** Univariate analysis of demographic and clinical variables by adenoma or polyp diagnosis

**2.a: Adenoma**	**Adenoma positive N = 300**	**Adenoma negative N = 956**	OR (95% CI)*	***P *** value*
Age ≥ 60 years, n (%)	129 (43)	295 (31)	1.7 (1.3-2.2)	<0.001
Male gender, n (%)	132 (44)	301 (32)	1.7 (1.3-2.2)	<0.001
*H. pylori* positivity, n (%)	108 (36)	258 (27)	1.5 (1.2-2.2)	0.003
Chronic active gastritis, n (%)	102 (34)	265 (28)	1.3 (1.0-1.8)	0.04
Baseline high risk, n (%)	8 (3)	13 (1)	2.0 (0.7-5.2)	0.1
**2.b: Polyp**	**Polyp positive N = 456**	**Polyp negative N = 800**	OR (95% CI)*	***P *** value*
Age ≥ 60 years, n (%)	169 (37)	255 (32)	1.3 (1.0-1.6)	0.06
Male gender, n (%)	177 (39)	256 (32)	1.3 (1.1-1.7)	0.015
*H. pylori* positivity, n (%)	158 (35)	208 (26)	1.5 (1.2-1.9)	0.001
Chronic active gastritis, n (%)	146 (32)	221 (28)	1.2 (1.0-1.6)	0.1
Baseline high risk, n (%)	13 (3)	8 (1)	2.9 (1.2-7.1)	0.014

*Unadjusted values.

In multivariate logistic regression, age (adjusted OR = 1.4 for each 10 years), male gender (adjusted OR = 1.7), and *H. pylori* positivity (adjusted OR = 1.5) were independent risk factors for colorectal adenoma (Table 3.a), with similar findings for colorectal polyps (Table 3.b).

**Table 3:** Multivariate logistic regression for predictors of colorectal adenoma or polyp

**3.a: Adenoma**	Adjusted OR (95% CI)*	***P *** **value**
Age (10 years)	1.4 (1.2-1.6)	<0.001
Male gender	1.7 (1.3-2.0)	<0.001
*H. pylori* positivity	1.5 (1.1-2.0)	0.003
**3.b: Polyp**	Adjusted OR (95% CI)*	***P *** **value**
Age (10 years)	1.2 (1.1-1.3)	0.005
Male gender	1.3 (1.0-1.7)	0.026
*H. pylori* positivity	1.5 (1.2-1.9)	0.002
Baseline high risk	2.9 (1.2-7.1)	0.021

Variables entered into each model were Age, gender, *H. pylori* status, chronic active gastritis, baseline high risk.

*OR are simultaneously adjusted for the rest of variables remained in the model.

### Pre-procedure’s indications as risk predictors for colorectal polyps

Using combination criteria of baseline high risk clinical presentations- such as lower GI blood loss, abdominal mass and/or family/personal history of colorectal polyps or cancer, was more sensitive and specific than using each presentation alone in the prediction of colorectal polyps, but not adenoma (Table 2). In multivariate logistic regression, baseline risk features were statistically significant predictors of colorectal polyps (adjusted OR [95% CI]: 2.9 [1.2-7.1]; p = 0.021).

### Serological assays for the detection of anti-*H. pylori* and anti-Cag-A

One hundred sixty three serum samples (including 81 polyp and 82 without polyps gender and age matched subjects) were analyzed for anti-*H. pylori* and anti-Cag-A antibodies. In these two groups, 85 subjects (45%) were positive for anti-*H. pylori*. Of these 85 sera, 60 (71%) were anti-Cag-A positive. The anti-*H. pylori* positive number in non polyp patients were 40 (49%) while they were 45 (56%) in the polyp patients’ sera (p = 0.3) while anti-Cag-A positivity was 73% in polyp patients vs. 68% in non polyp controls (p = 0.5). Thirty three (55%) patients who were positive for both *H. pylori* and Cag-A had polyps while 33 (47%) patients of those negative for both *H. pylori* and Cag-A had polyps (p = 0.3; Table 4). The corresponding figure for those positive for *H. pylori* and negative for Cag-A was 12 (48%). While the p values were not statistically significant, the observed pattern of higher polyp prevalence in *H. pylori*/Cag-A patients is consistent with the overall epidemiological results.

**Table 4:** **Serological testing for anti-*H. pylori *** **(HP) and anti-Cag-A in patients with polyps**

***H. pylori* status**	**Number of patients**	**Polyps**	OR (95% CI)*
HP Negative	70	33 (47%)	1
HP Positive			
HP Ab + & Cag Ab +	60	33 (55%)	1.37 (0.65-2.90)
HP Ab + & Cag Ab –	25	12 (48%)	1.03 (0.37-2.85)
HP Ab - & Cag Ab +	8	3 (38%)	0.67 (0.10-3.79)
Total	163	81 (50%)	

*Unadjusted.

## Discussion

Our study indicates that gastric *H. pylori* infection, confirmed by immunohistochemistry staining of gastric biopsies, associates with an increased risk of colorectal lesions in African Americans. The same association was true in 3 meta-analyses which included studies from different ethnic groups. In particular, Zumkeller et al. [45] included 11 studies regardless of the diagnostic tests used for *H. pylori,* and Zhao et al. [13] included 10 out of 13 case–control studies that used only IgG antibody for *H. pylori* to demonstrate previous *H. pylori* infection in patients with colonic lesions. Jones et al. [46] used immunohistochemistry methods to demonstrate that *H. pylori* do reside in the subjects’ colon biopsies and associates with colorectal neoplasm.

Our previous report showed colorectal polyps’ incidence begins to increase significantly at age forty in African Americans [47] with or without family history of CRC, thus it has been chosen as a cutoff age in this study. Aging male patients are at a higher risk of adenomatous and hyperplastic polyps [48], and are more likely to associate with *H. pylori* positivity which is consistent with findings in other ethnic groups [49]. In this study, we were not able to establish a correlation between histopathological subtypes, size, and location of colorectal polyps with *H. pylori* infection.

Other confounders such as BMI, smoking, alcohol consumption, diabetes, socioeconomic status, diet and lack of physical activity are known risk factors for colorectal polyp/cancer. Our retrospective epidemiological study did not adjust for these factors because of lack of corresponding information. Previous studies reported an association between *H. pylori* and colorectal adenoma which was not altered even after adjustment for confounding factors [14,50].

To our knowledge, no previous study has linked *H. pylori* infection with colorectal polyps or cancer in African Americans. The higher prevalence of colorectal polyps in *H. pylori* infected patients and the higher seropositivity could be explained by their environmental and genetic differences as well. Potentially, such differences may alter host gastric and/or colorectal mucosa in response to *H. pylori* carcinogenic effect among some ethnic groups. For instance, Indians have a low rate of gastric cancer and high rate of *H. pylori* infection which was not significantly associated with intestinal metaplasia, gastric tumor site, and patient’s age [51]. Moreover, despite the high prevalence of gastric cancer in Finland, there was no association between atrophic gastritis or *H. pylori* infection with colorectal cancer among Finnish male smokers [19,52].

High risk features when combined at presentation were more predictive for colorectal polyps, As such, they can be useful clinical criteria to prioritize access to colonoscopy. The risk prediction of colorectal polyps based on gastric lesions recovered from gastroscopy depend on the nature of such lesions. The highest prevalence of colorectal polyps among *H. pylori* positive subjects was found among those who had chronic active gastritis. Such polyps were more likely to be neoplastic (adenomatous). Due to the retrospective nature of the study, the time lapse between the *H. pylori* infection and the colonic lesions occurrence is unknown. Meira et al. [8] reported after infecting mice with *H. pylori*, the chronic inflammation induced DNA damage in alkyladenine DNA glycosylase deficit mice and enhances inflammation-associated colon tumorigenesis. It also predisposed to the development of gastric cancer precursor lesions.

As gastric lesions progressed, *H. pylori* positivity decreased and the risk of colorectal polyps increased, being highest in patients with chronic atrophic gastritis with intestinal metaplasia, but the number of associated colorectal polyps was too small to make meaningful conclusions. The annual progression from chronic non-atrophic gastritis to atrophic gastritis is 1 to 3% [53,54]. *H. pylori* in advanced gastric lesions has probably decreased because of migration through the gastrointestinal tract [55]. This suggests that with advanced gastric lesions, gastric lesions but not *H. pylori* status would be appropriate in the prediction of colorectal polyps [23,56,57]. Bulajic et al. [24] found only 1.2% of malignant colorectal tissues were positive for *H. pylori* in contrast to 6% positive normal tissues from cancer patients. This could be explained by migration phenomena too [46] and would suggest that *H. pylori* gastric effect could be similar to its colonic effect. Once *H. pylori’*s infection is established, it likely elicits robust and lasting inflammatory and immune responses that may potentially influence the development of diseases that occur later in life such as cancer.

The strengths of our study; are in its comprehensive nature since it linked the real time presence of gastric *H. pylori*, the symptoms it may produce, and the associated gastric lesions with its extra-gastric colonic effects [58]. Gold standard tools were utilized to diagnose colorectal polyps (complete colonoscopy) and *H. pylori* infection (gastric biopsy). We used immunohistochemistry to diagnose *H. pylori* in gastric biopsies as it is highly sensitive and specific particularly in patients who have been partially treated. Besides, we had a fairly large sample size (n = 1256). Moreover, the findings with this large sample were further confirmed serologically.

Because our study was retrospective and hospital-based rather than population-based, it has its own limitations. The underestimation of *H. pylori’s* prevalence and its associated gastric lesions in the prediction of colorectal polyps could be explained by gastric sampling errors and unknown received treatments. Also, our serology testing sampling was smaller (n = 163) than the sampling for the epidemiological study (n = 1256). Despite that, we were able to prove positive association between *H. pylori* and colorectal polyps consistent with the colon cancer mouse models studies [58]. Also, we were able to show that there is a trend of increased chance of having polyps in the presence of Cag-A positive *H. pylori* infections.

## Conclusions

In conclusion, forty years and older African Americans with gastric *H. pylori* infection were at high risk of neoplastic and non-neoplastic colonic lesions. *H. pylori* associated chronic active gastritis and alarming clinical features at presentation may necessitate early screening colonoscopy and/or *H. pylori* eradication. Prospectively designed studies are needed to establish the conditions in which the current *H. pylori* infection in gastric mucosal lesions might participate in the colon carcinogenic transformation, either through its colonization of the colon and/or through its metabolites and whether the eradication of *H. pylori* would reduce colon polyp incidence in African Americans in the future.

## Abbreviations

AA: African Americans; CRC: Colorectal cancer; IHC: Immunohistochemistry; GI: Gastrointestinal.

## Competing interests

The authors declare they have no competing interests.

## Authors’ contributions

MZ and HB contributed equally in acquisition and interpretation of the data, EL contributed in pathology interpretation of results, DS and AOL contributed in sample recruitment, MN and HR performed the statistical analysis, GPP performed H. pylori Cag-A analysis, and HA designed and wrote the paper and submitted the manuscript for publication. All authors read and approved the final manuscript.

## Authors’ information

HA is the director of microarray lab at Howard University Cancer Center.

## Pre-publication history

The pre-publication history for this paper can be accessed here:

http://www.biomedcentral.com/1471-2407/14/296/prepub
